# Study on Magnetic Control Systems of Micro-Robots

**DOI:** 10.3389/fnins.2021.736730

**Published:** 2021-08-26

**Authors:** Youjia Shao, Ashraf Fahmy, Ming Li, Chunxu Li, Wencang Zhao, Johann Sienz

**Affiliations:** ^1^School of Automation and Electronic Engineering, Qingdao University of Science and Technology, Qingdao, China; ^2^Faculty of Science and Engineering, Swansea University, Swansea, United Kingdom; ^3^Faculty of Engineering, The Chinese University of Hong Kong, Shatin, Hong Kong SAR, China; ^4^Department of Electrical Power and Machines, School of Engineering, Helwan University, Helwan, Egypt; ^5^Centre for Robotics and Neural Systems, University of Plymouth, Plymouth, United Kingdom

**Keywords:** micro-robot, magnetic control system, electromagnet, permanent magnet, magnetic field

## Abstract

Magnetic control systems of micro-robots have recently blossomed as one of the most thrilling areas in the field of medical treatment. For the sake of learning how to apply relevant technologies in medical services, we systematically review pioneering works published in the past and divide magnetic control systems into three categories: stationary electromagnet control systems, permanent magnet control systems and mobile electromagnet control systems. Based on this, we ulteriorly analyze and illustrate their respective strengths and weaknesses. Furthermore, aiming at surmounting the instability of magnetic control system, we utilize SolidWorks2020 software to partially modify the SAMM system to make its final overall thickness attain 111 mm, which is capable to control and observe the motion of the micro-robot under the microscope system in an even better fashion. Ultimately, we emphasize the challenges and open problems that urgently need to be settled, and summarize the direction of development in this field, which plays a momentous role in the wide and safe application of magnetic control systems of micro-robots in clinic.

## Introduction

Micro-robot is a typical micro-electromechanical system, which refers to a type of robot with a scale of 1 micron to a few millimeters and is capable of autonomous movements (Nelson et al., 2010; Sitti et al., 2015). With the cross-development of disciplines such as microelectronics, cybernetics, computers, materials and bionics, it has brought opportunities for rapid development of micro-robot technology in the medical field, promoting automation and robotization in the medical field (Schmidt et al., 2020; Yang et al., 2020b). There are four main applications in medical treatment. One is to clear the cardiovascular blockage. The second is to destroy the local lesions of the human body, and the third is to realize the diagnosis of the disease. The fourth is precise drug deliver. When the size of the robot is reduced to the micron or nanometer level, the inertial force of the robot is negligibly small and the resistance of the liquid plays a major role. Human body fluids are located in a low Reynolds number (Re) environment (Zhang et al., 2009; Ng et al., 2019; Xu et al., 2019; Yu et al., 2019). When the Reynolds number is low, either the viscosity of the liquid is high, or objects in this environment move very slowly, or the size of the object is small (Abbott et al., 2009). Due to the different working environments, the micro-robot needs to use a distinct driving method than the large robot to keep pushing.

At this stage, this paper mainly studies magnetic field driving method of micro-robots. Magnetically controlled micro-robots, which can be dynamically adjusted by the external driving magnetic field to take action on the permanent magnet inside the robot, can obtain the desired driving force and auxiliary torque (Li et al., 2018; Yu et al., 2018). This exterior magnetic field driving method can enhance the flexibility and increase the moving distance of the micro-robot in the body. What's more, there is no limit to the walking route, which can be accurately controlled in real time. Magnetic field driving has significant advantages, with controllability and safety (Gang et al., 2011; Yan et al., 2015). In the medical field, some traditional actuation methods, such as endoscopes or catheters, can be inserted into the body from the mouth or anus of patients. However, this will make the patient feel a strong sense of discomfort during the detection and these traditional methods have working blind spots as well as collected patient information is not comprehensive (Yan et al., 2017). By comparison, micro-robots driven by external magnetic fields can perform a full range of inspections in the human body, and they will not cause too much discomfort to humans with high precision and efficiency (Yu et al., 2017). These micro-robots can better adapt to the narrow working space and internal environment, and are expected to become the mainstream development trend of micro-robots. At present, the magnetic field driving method is a generally recognized functional method in the field of micro-robot driving, having a broad application prospect (Lee et al., 2001; Jin et al., 2016; Yang et al., 2017, 2020a; Wang et al., 2018).

Even though magnetic control systems of micro-robots have made great progress, they still have some instability and most of them are currently limited to research in the laboratory environment. In order to obtain a better observation of the movement of micro-robots under the microscope in the laboratory, we utilize SolidWorks2020 software to partially change and model the SAMM system (Wright et al., 2017). The optimization of magnetic control systems for entering the clinic widely and safely has become inevitable, and it is also the focus of the further promotion of micro-robots. In this paper, we firstly introduce principles of magnetic navigation and background. Secondly, we mainly focus on reviewing magnetic control systems with classification and analyses in three types. Thirdly, we provide mechanical design of spherical permanent magnet system and point out the challenges and open problems. We finally conclude this paper.

## Background

### Principles of Magnetic Navigation

**Essence of Magnetic Phenomena** When micro-robots embedded with permanent magnets or containing magnetic materials are placed in a magnetic field, they interact with external magnetic fields (Abbott et al., 2020). Generally speaking, the micro-robot is called magnetic medium and can respond to external magnetic field. Whether it is the magnetic field generated by the electromagnet or permanent magnet, the essence of the magnetic phenomenon comes from the current or the moving charge (Zhang et al., 2017).

Magnetic Force and Moment Owing to the small size of the micro-robot, it can be regarded as a magnetic dipole model relative to the external magnetic field (Song et al., 2017). Let ***m*** denote the magnetic moment of the magnetic dipole and ***B*** denote magnetic flux density, then the potential energy of the magnetic dipole in the external magnetic field is (Nguyen et al., 2016):

(1) $$\begin{array}{l} U=-\text{m}\cdot \text{B} \end{array}$$

Using law of conservation of energy (Sarton et al., 1929), the magnetic force on the magnetic dipole can be obtained as (Boyer, 1988):

(2) $$\begin{array}{l} \text{F}=-\nabla U=-\left(\text{m}\cdot \text{B}\right) \end{array}$$

According to Maxwell's equation (Monk, 1992), ∇ ·***B*** = 0, then formula (2) becomes:

(3) $$\begin{array}{l} \text{F}\;=\;\nabla \text{B}\;\cdot \;\text{m} \end{array}$$

It can be viewed in formula (3) that the direction of the magnetic dipole in the external magnetic field is consistent with the direction of the gradient of magnetic flux density, and its magnitude is proportional to the magnitude of the gradient of magnetic flux density. Furthermore, it can be known that the magnetic dipole is only subjected to magnetic force under the condition in which the external magnetic field has a gradient instead of a uniform magnetic field. The magnetic moment of the magnetic dipole in the external magnetic field can be expressed as (Vaidman, 1990):

(4) $$\begin{array}{l} \text{T}\;=\;\text{m}\;\times \;\text{B} \end{array}$$

It can be seen that when the magnetic moment ***m*** and the external magnetic field ***B*** form an angle of 90 degrees, the magnetic moment reaches the maximum. When the magnetic moment ***m*** is parallel to the magnetic field ***B***, the magnetic moment is zero (Sun et al., 2016). Therefore, the direction of a magnetic dipole would align to the external magnetic field and a rotating magnetic field would generate rotating movements of the magnetic micro-robot. Generally speaking, the magnetic moment controls the orientation of the micro-robot, and the magnetic force controls the total force acting on the micro-robot, thereby controlling the position of the micro-robot (Xu et al., 2015).

### Historical Background

In the medical field, micro-robots can not only assist doctors to complete surgical operations, but also achieve high precision and quality of surgery while avoiding damage (Yang and Zhang, 2020a). The micro-robot can not only improve the safety of the operation and eliminate side effects, but also greatly shorten the treatment time. The application of micro-robots in the medical field can also relieve strained medical resources and reduce the psychological pressure of doctors during surgery. For driving methods of micro-robots, they have experienced the following (Kortschack et al., 2003). One is the traditional endoscope, which is inserted into the body directly from the outside by the flexible tube guide. The second is gastrointestinal peristalsis-promoting micro-robot. The third is the self-creeping micro-robot, which promotes movements of the micro-robot in the body fluid through the earthworm-type self-perturbation mode. The above several driving methods contain the disadvantages of human damage, low efficiency, non-real-time control and low flexibility, and external magnetic field driving methods that can avoid these shortcomings have gradually begun to develop. The magnetic field driving method was first developed from stationary electromagnet control systems and permanent magnet control systems. The former uses a combination of a variety of fixed electromagnetic coils to control micro-robots, the typical ones are OctoMag (Kummer et al., 2010) and MiniMag (Kratochvil et al., 2014) systems. The latter generally utilizes movable external permanent magnets to form a magnetic field to control the movement of the micro-robot. The external permanent magnet is generally fixed on the robotic arm. After a period of development of these magnetic control systems, a new type of control system appeared, which is the mobile electromagnet control system. This system is a combination of the above two, combining the strengths of the two control systems, and it can use movable electromagnetic coils to form an external magnetic field (Chen et al., 2017). Through the above development, these three basic branches of magnetic control systems applied to the micro-robot are formed.

## Taxonomy

So far, many papers have introduced different magnetic control systems. Through our own summary and analyses, we have simply divided them into three types: the stationary electromagnet control system, the permanent magnet control system and the mobile electromagnet control system. Partial existing magnetic control systems are reviewed.

### Stationary Electromagnet Control Systems

**Figure 1:**
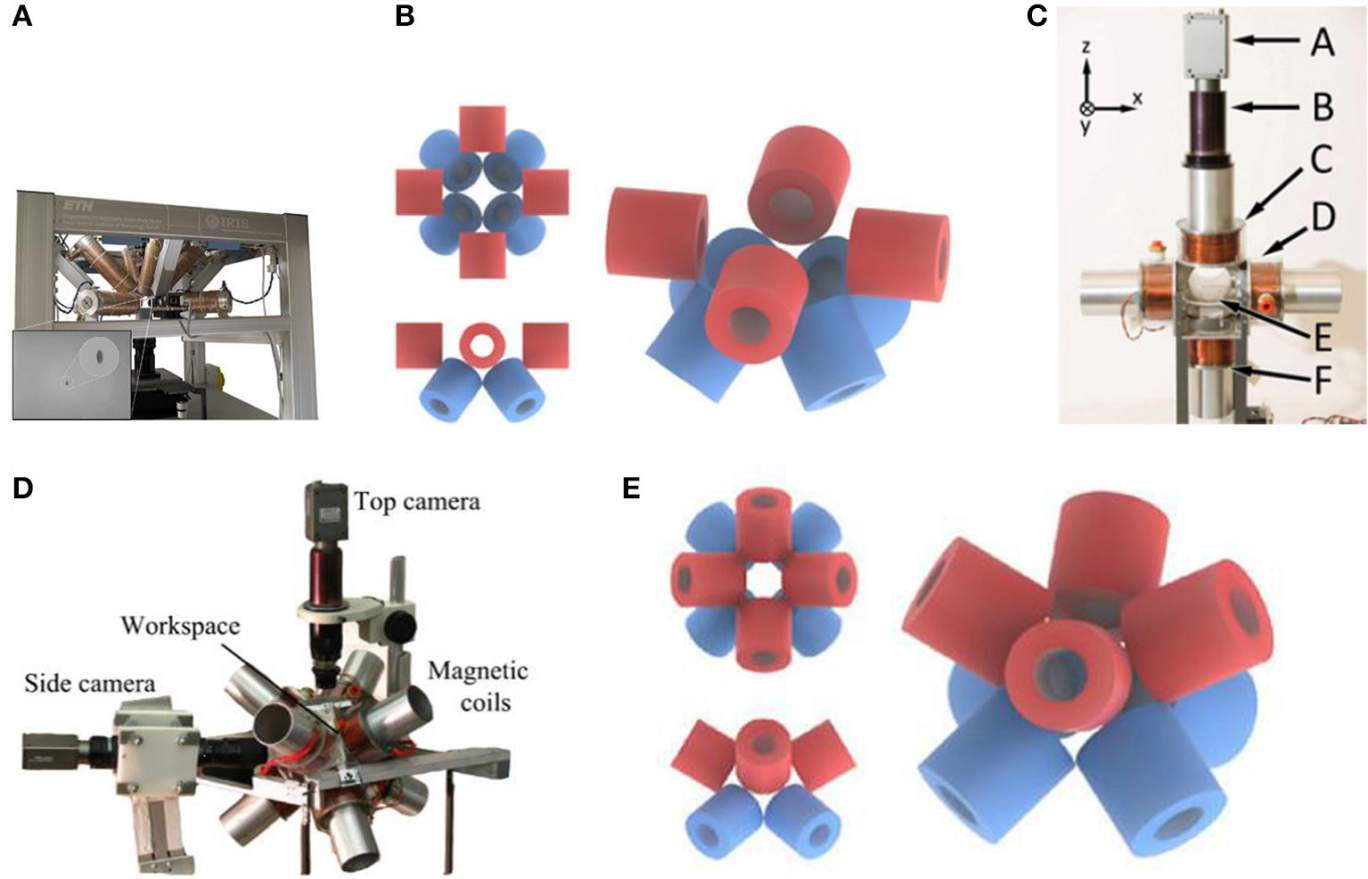
**(A)** OctoMag control system has eight fixed electromagnetic coils for 5-DOF control (Kummer et al., 2010). **(B)** Model of electromagnetic coils configuration of OctoMag shows that the upper electromagnets have rotated 45 degrees relative to the lower electromagnets (Pourkand and Abbott, 2018). **(C)** Six electromagnetic coils setup with a camera for top-view vision feedback and a microscope lens on the top for controlling Mag-μBots (Diller et al., 2012) **(D)** Eight electromagnetic coils system to control multiple micro-robots with different structures (Diller et al., 2013) **(E)** Model of electromagnetic coils configuration shows that 8-electromagnetic coils are distributed diagonally (Pourkand and Abbott, 2018).

In 2010, Kummer et al. (2010) proposed a stationary electromagnetic system called OctoMag for 5-degree-of-freedom (DOF) wireless micro-manipulation, as shown in Figure 1A. This system is designed to be used in retinal surgery. Because retinal vein cannulation is very difficult, in order to achieve safer and more advanced control, the micro-robot freely controlled by the OctoMag system has become a breakthrough. The OctoMag system uses a linear representation of multiple magnetic fields in a complex non-uniform magnetic field to perform 5-DOF magnetic control of the micro-robot, including 3-DOF position and 2-DOF pointing orientation. They selected eight fixed electromagnets to form the entire OctoMag system, while the upper group of electromagnets rotated 45 degrees relative to the lower group. OctoMag electromagnetic coils modeling combination configuration is shown in Figure 1B (Pourkand and Abbott, 2018). The electromagnet system is also equipped with a cooling system around each coil. The operator can adjust the position using only visual feedback without position feedback. The system only implements open-loop orientation control and closed loop position control. However, the system can accurately control the error between the expected orientation and the actual orientation of the micro-robot, especially when the position is known. It can be scaled down to control micro devices in optical microscopes and spiral magnetic cursors. This single magnetic control system can be equivalent to the foundation of a complex system, and can be innovated and modified in different ways to achieve new applications. However, for surgical operations that require greater force, it is not the best choice.

In 2012, Diller et al. (2012) have made new progress in the research of controlling multiple magnetic micro-robots. These magnetic micro-robots are called Mag-μBots, each of which is within 1 mm in size. The external magnetic field system provides magnetic braking, as shown in Figure 1C (Diller et al., 2012). Different from 8 electromagnetic coils of OctoMag (Kummer et al., 2010), this magnetic control system consists of six independent air-core electromagnetic coils, with a camera for top-view vision feedback and a microscope lens on the top, forming a 20 × 20 mm working space in the center of the coils system. The researchers conducted experiments on a set of Mag-μBots with different geometric shapes and a set of Mag-μBots with the same geometric shapes. Since the response of each robot to the control signal of the braking magnetic field is unique, by learning the speed response of each micro-robot to the control signal of different frequencies, each Mag-μBots can be independently one-dimensional and two-dimensional controlled on a non-special operating surface. Even Mag-μBots of the same shape have slight differences. Experiments show that at present, only independent control of up to three micro-robots can be achieved. If people want to perform heterogeneous control of more than three Mag-μBots, it also needs to reduce the coupling between robots and improve the accuracy of learning speed response. The researchers will make further efforts in path planning and control algorithms in the next step to achieve the three-dimensional control of Mag-μBots and complete the task of manipulating objects.

OctoMag only shows one combination of eight electromagnetic coils, and there are also other control systems for the combination of eight electromagnetic coils. In 2013, Diller et al. (2013) proposed the independent control of multiple magnetic micro-robots in three dimensions using electromagnetic coils system for implementation. Researchers determine that, to achieve independent control of multiple micro-robots in 3D space, it is necessary to design micro-robots with different structures and create unique phase lags for each micro-device. They use micro-robots with different structural geometry or magnetic properties to perform different movements. In this regard, they use a magnetic control system composed of eight electromagnetic coils to complete the magnetic manipulation. The system structure is shown in Figure 1D (Diller et al., 2013), and the eight electromagnetic coils are diagonal-distributed. The specific modeling configuration of the electromagnetic coils is shown in Figure 1E (Pourkand and Abbott, 2018). The system is imaged by two cameras and a microscope lens, and the current input is monitored by a remote computer platform as well as Hall Effect sensors. Through the external magnetic manipulation of the experiment, multiple micro-robots with different structures in the fluid can be subjected to different magnetic forces and can be independently controlled. However, this method still has defects. When the number of controlled micro-robots increases, the control ability will be weakened.

**Figure 2:**
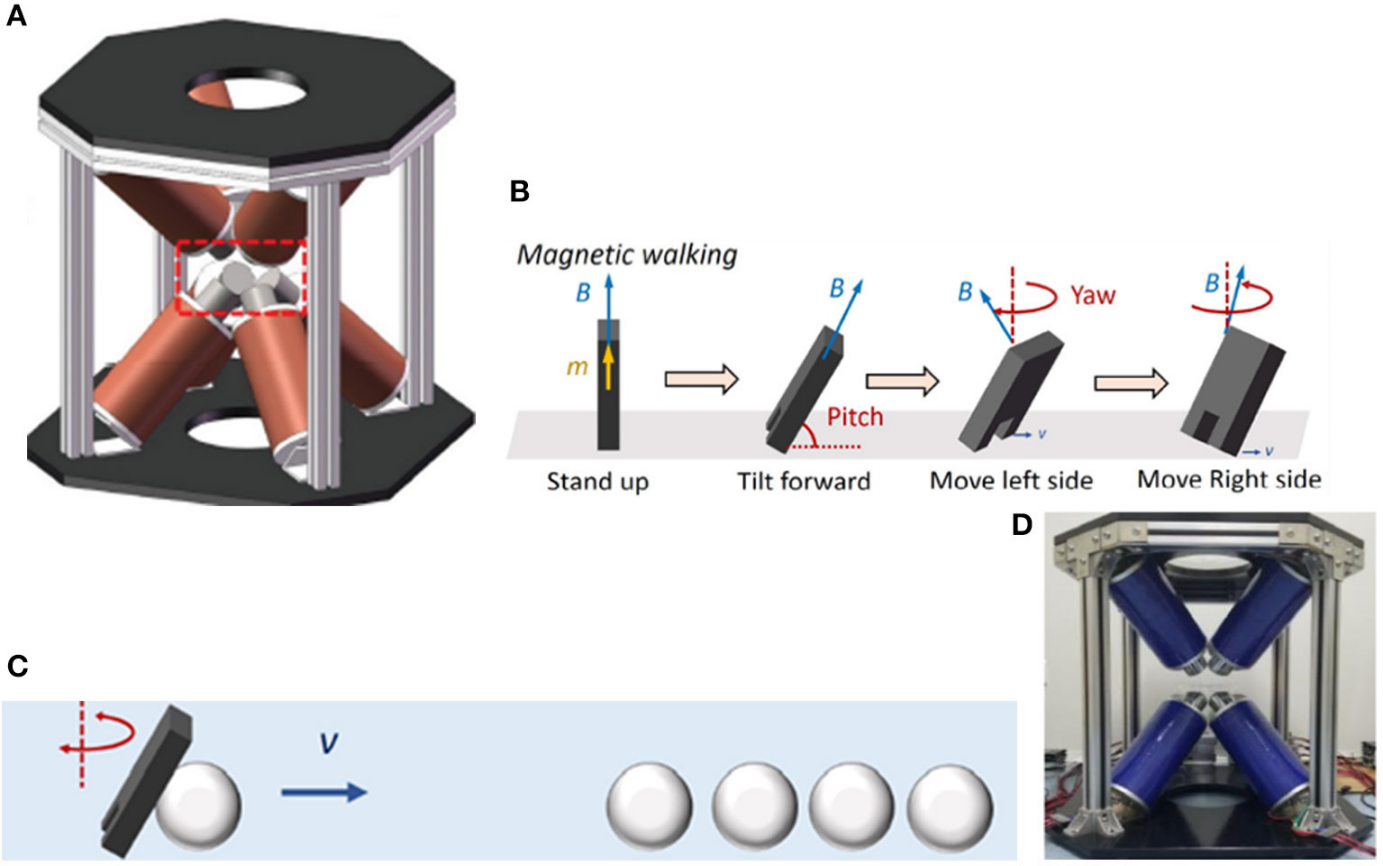
**(A)** External electromagnetic system has 8 electromagnetic coils distributed diagonally to control a micro robot with two pseudopods to perform magnetic walking (Li et al., 2020). **(B)** The process of magnetic walking of a micro-robot by alternately raising the left and right feet (Li et al., 2020) **(C)** Indirect pushing of non-magnetic microbeads (Li et al., 2020) **(D)** Octupole magnetic system provides magnetic drive in rotating field and gradient field to ISMEs (Zheng et al., 2021).

In 2019, Li et al. (2020) designed a new type of micro-robot. This robot has two pseudopods, similar to human feet, which can be alternately lifted and moved. In this experiment, the electromagnetic system is used to manipulate the micro-robot so that it can complete magnetic walking. As shown in Figure 2A (Li et al., 2020), this magnetic field control system has the same structure as the coil system proposed by Diller et al. (2013), with 8 electromagnetic coils distributed diagonally. The specific modeling configuration of this electromagnetic coils is shown in Figure 1E (Pourkand and Abbott, 2018). The magnetic system with the same coil structure can carry out the research of a variety of micro-robots, but robots with different structures and shapes have different requirements for the magnetic field provided by the control system. This 8-electromagnetic coil system provides a periodic magnetic field, and the oscillation waveform, amplitude and frequency of the magnetic field are designed in advance according to the planned path. The micro-robot will alternately lift its two feet to produce displacement according to its own gravity and friction in this magnetic field, as shown in Figure 2B (Li et al., 2020). Non-magnetic objects can be manipulated indirectly through magnetic walking by the robot, such as indirect pushing of non-magnetic microbeads, as shown in Figure 2C (Li et al., 2020). This shows that the micro-robot can achieve indirect magnetic manipulation under a periodic magnetic field, which has huge research potential.

In 2021, Zheng et al. (2021) designed ionic shape-morphing micro-robotic end-effectors (ISMEs) that can be used for environmental targeting, releasing and sampling, and have a gripping motion. The magnetic nanoparticles are encapsulated in an alginate monolayer, and the ISMEs are controlled by the external magnetic field to reach the target position. This magnetic system is controllable and provides the ISMEs with a rotating magnetic field and a gradient magnetic field. The system consists of 8 electromagnetic coils with DT4 cores, which are distributed diagonally. The structure of this coil system is the same as that used by Diller et al. (2013) and Li et al. (2020), as shown in Figure 2D (Zheng et al., 2021). The specific modeling configuration of this electromagnetic coils is shown in Figure 1E (Pourkand and Abbott, 2018). However, the controlled micro-robots and the completed operations are different, and the details and control characteristics of the magnetic control system also have differences. A micro-camera and a lens with a focal length of 25 mm constitute an imaging system and the current of each coil is individually controlled through an amplifier. Due to the pH change in the liquid environment and the adjustment of the ionic solution, the ISMEs achieve contraction and grip, and internal magnetic balls are separated from the ISMEs to complete the drug delivery. Micro-magnetic spheres can be excreted from the body through excretion function, and will not produce toxicity. ISMEs can also be propelled and navigated through internal magnetic nanoparticles to achieve sampling functions. Researchers believe that these ISMEs have great potential in medical applications such as targeted therapy and precise tissue diagnosis.

**Figure 3:**
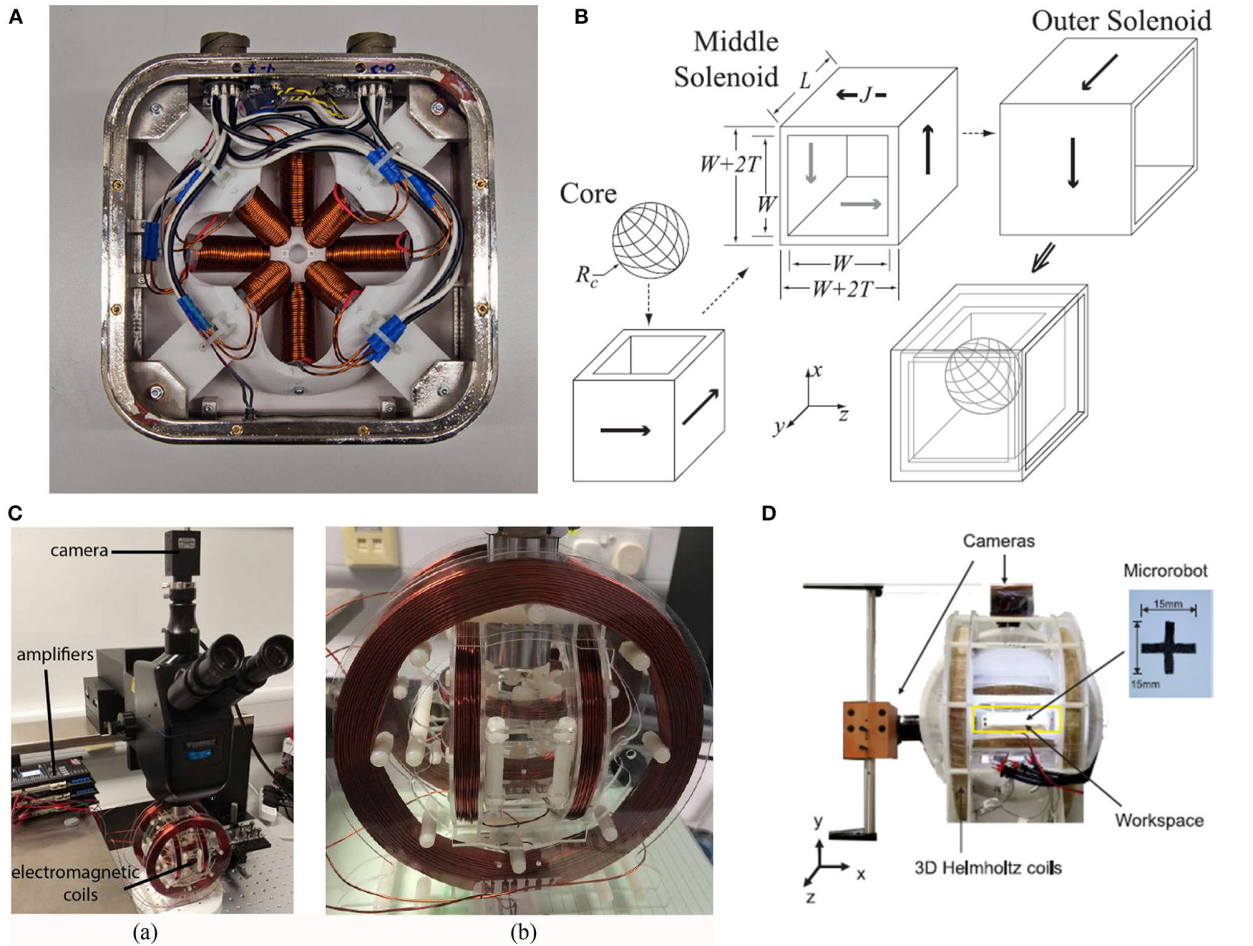
**(A)** MiniMag control system restricts 8 electromagnetic coils in a fixed hemisphere for the control of micro robots with a small working distance from the system (Kratochvil et al., 2014). **(B)** Omnimagnet control system has three nested cubic electromagnetic coils and magnetic ball inside (Petruska and Abbott, 2014). **(C)** Three-axis Helmholtz electromagnetic coils system with a PointGrey camera and three Maxon motor controllers are used for generating the dynamic magnetic fields (Yu et al., 2017). **(D)** 3D helmholtz coils system with two cameras for controlling CTM (Su et al., 2020).

In 2014, Kratochvil et al. (2014) proposed a hemispherical magnetic steering system for 5-DOF control, called MiniMag. The MiniMag system is similar to the previous OctoMag system and consists of eight stationary electromagnetic coils with soft magnetic cores (Kummer et al., 2010). OctoMag system is mainly for the operation of the system with a larger working distance, while MiniMag is mainly for the smaller, using a 10 mm spherical working space, as shown in the Figure 3A (Kratochvil et al., 2014). MiniMag restricts the position of eight electromagnets in a fixed hemisphere. Since the tilted electromagnets occupy a hemispherical shape, it can be physically combined directly with the microscope system. The thickness of the MiniMag coil is thinner and the working space is larger than OctoMag, and the system uses the center point setting in the workspace to lower the need for closed-loop feedback when the workspace is small. MiniMag is essentially another form of OctoMag. All eight electromagnets are in the linear limit and can be modeled separately. MiniMag system can be applied under an optical microscope and perform cell manipulation under high-resolution optics. The system has advantages in controlling micro-robots with different modes in a tiny space.

In 2014, Petruska and Abbott (2014) proposed an omnidirectional electromagnet capable of controlling the generation of dipole fields, called Omnimagnet. The omnidirectional electromagnet combines the real-time control of the traditional electromagnetic system and the rotating dipole field of the permanent magnet to generate a magnetic field with a variable dipole moment, avoiding motion damage. Omnimagnet consists of three nested cubic electromagnetic coils and magnetic ball inside. The assembled omnidirectional electromagnet is shown in Figure 3B (Petruska and Abbott, 2014). The radius of the magnetic ball is 50 mm, and it is easy to be magnetized. Omnimagnet provides independent inputs for the three helical coils to generate magnetic fields and magnetic field gradients at every point in the workspace. A single Omnimagnet can achieve 3-DOF control. By adding a cooling system to this system for valid application, as the size increases, the dipole strength will also increase. In this regard, the researchers also pointed out that combining Omnimagnets of different shapes can form more complex magnetic control systems to achieve different degrees of freedom control, such as the aforementioned OctoMagnet (Kummer et al., 2010) and MiniMagnet (Kratochvil et al., 2014). It is undeniable that Omnimagnet has broad prospects for future magnetron applications.

In 2017, Yu et al. (2017) proposed to use an external dynamic magnetic field to decompose paramagnetic nanoparticle chains. The magnetic field is pre-designed and the whole process is controllable. The decomposed chains reduce the chance of recombination by increasing the distance between each other, making the decomposition process more stable. At the same time, the length and speed of the chain can be adjusted in a restricted environment. The shortened chain facilitates the effective transportation of goods through narrow areas and achieves controllable performance. The magnetic field control system used in the experiment is shown in Figure 3C (Yu et al., 2017). It is composed of three-axis Helmholtz electromagnetic coils, and is equipped with a PointGrey camera on the top to record the experiment and three Maxon motor controllers as amplifiers, which can provide rotating magnetic field in the workspace. The magnetic field achieves decomposition through the diffusion and fragmentation of the particle chain, and the assembly process can also be carried out reversibly. Researchers use the phase lag model to verify the experimental results, which is of great significance to the development of micro-robot groups.

In 2020, Su et al. (2020) proposed a soft cruciform thin-film micro-robot (CTM), which has two motion modes, jellyfish-like mode and forklift mode. Since the micro-robot is soft, it will reduce the damage to the cells and molecules during operation. CTM is magnetic and controlled by an external magnetic field. As shown in Figure 3D (Su et al., 2020), the control system is composed of 3D Helmholtz coils, which is the same as the coil system structure used in the previous decomposition of paramagnetic nanoparticle chains (Yu et al., 2017). The orthogonal combination of three pairs of coils provides a precise and controllable external magnetic field and the working space is 90 × 90 × 40 mm. However, the function and structure of the controlled robot are different from the above mentioned system (Yu et al., 2017). Some characteristics of the magnetic field, including current configuration, imaging components, and control algorithms are also different. The magnetic field system has two cameras to feed back the control picture to the monitoring screen in time. The CTM controlled by this magnetic system moves in an S-shaped trajectory, and the moving speed is proportional to the frequency of the magnetic field, and also proportional to the length and thickness of the robot's legs. Experiments show that the weight of objects that CTM can carry is 10 times its own weight, and it has the ability and potential to deliver drugs to target locations in the medical field.

**Figure 4:**
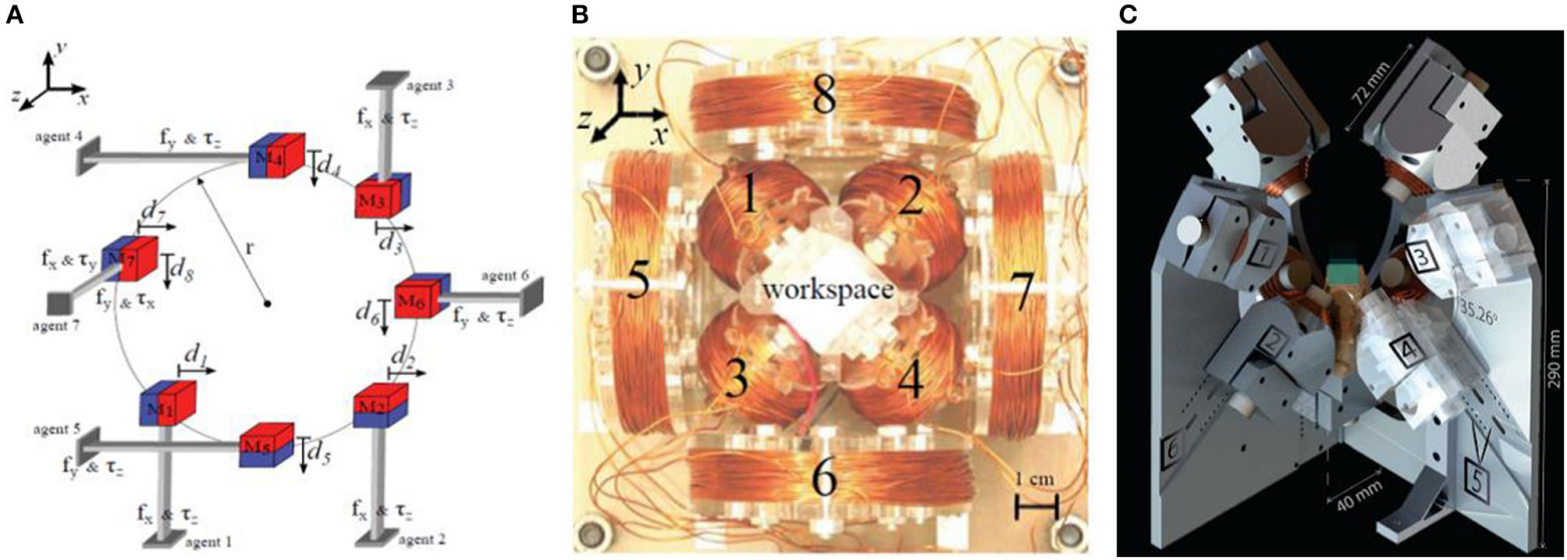
**(A)** Seven permanent magnets proof mechanism system is used to prove that the maximum 8-DOF magnetic control can be achieved (Salmanipour and Diller, 2018). **(B)** Magnetic field generation prototype for 8-DOF control, consisting of four inner coils and four outer coils (Salmanipour and Diller, 2018) **(C)** BatMag control system has 9 electromagnetic coils for 6-DOF control (Ongaro et al., 2019).

In 2018, Salmanipour and Diller (2018) proposed an 8-DOF remote actuation of small magnetic mechanisms. Most magnetic field control systems have achieved 5-DOF or 6-DOF control. Based on the premise that the external magnetic field is uniform in the working space, the researchers analyze the magnetic field components from a single point in the working space and adjust the magnetic control signal. But in this work, Salmanipour and Diller showed that under certain conditions, the highest independent 8-degree-of-freedom control can be achieved. In the magnetic micro-robot system, through the derivation of the formula concept, the input of the system is always the same 8 × 1 vector. The maximum number of independent outputs cannot be >8 inputs. A simple system is designed to verify up to 8-DOF for remote controlling. The proof mechanism system includes 7 cubic permanent magnets, and each permanent magnet can only move a small distance along one or two axes, with a total of 8-DOF, as shown in Figure 4A (Salmanipour and Diller, 2018). To control eight items, a magnetic drive system composed of 8 electromagnetic coils is designed. Eight electromagnetic coils with different directions are placed around the workspace to independently control the current input, as shown in Figure 4B (Salmanipour and Diller, 2018). In this work, researchers have verified the maximum possible DOF is 8, and designed an 8-DOF millimeter-level magnetic mechanism to verify the capability. But after all, this is just a conceptual theoretical verification. They believe that future work will focus on applying the theory to the magnetic control of micro-robots to express practicality.

In 2018, Ongaro et al. (2019) proposed an electromagnetic control device. The system can independently control the same and distinct micro-robots in 3D space with 6 degrees of freedom, which is called BatMag. Compared with OctoMag (Kummer et al., 2010) and MiniMag (Kratochvil et al., 2014), BatMag intuitively increases to 6-DOF control. Researchers use combined electromagnetic coils to generate a strong magnetic field and a magnetic field gradient, which can form the interaction force of gravity. BatMag is finally designed as a combination of nine stationary electromagnetic coils and the inner core of every coil is a permanent magnet. The overall system is shown in Figure 4C (Ongaro et al., 2019). A thermal management method was designed, using a water-cooling system to transfer the heat from the coil to the aluminum radiator. Meanwhile, researchers have developed and verified related algorithms that can be followed of the micro-robot. Utilizing the inhomogeneity of the generated magnetic field, BatMag system can independently control different and identical micro-robots with maintaining flexibility and high controllability. It is also considered to have significant potential in controlling clinically compatible imaging applications or controlling micro-robot clusters.

For the stationary electromagnet control system, the static electromagnetic coils can control the field strength by quickly changing the coil current, and freely turn on or off the exterior magnetic field, which has certain advantages. The electromagnetic system has sufficient flexibility in the controlling process and can change the magnetic field through diverse current inputs. Under the condition of unsaturation, the input relationship between the magnetic field and the current is linear (Lee et al., 2004). It is worth noting that hollow-core electromagnets are more conducive to model and control than core electromagnets, but the magnetic field generated is relatively weak. In the case of multiple coils, the combined magnetic field can be decoupled. The magnetic field generated by a single coil is calculated separately, and superimposed using a linear method. Meanwhile, the electromagnetic coil also has its specific limitations. Awaited to Joule heating when the power is turned on, the temperature of coils will increase, and a cooling scheme is usually required. However, because of conservation of energy, the cooling scheme will cause the temperature of the working environment to rise, which will influence the control of the robot and cause errors. In addition, owing to the relatively large distance between the field source and the controlled object, electromagnetic coils should provide a strong magnetic field, which will cause poor flexibility and difficulties in performing a large-scale operation. Increasing the volume of the system is usually by changing the size, number or distribution structure of coils, which will affect the input of the current, and the input calculation model will have to be re-planned, thereby affecting the control function of the entire system. At the same time, once the cored electromagnet reaches saturation, the above input relationship will become non-linear, which will affect the modeling and control of the coil (Yang and Zhang, 2020b).

### Permanent Magnet Control Systems

**Figure 5:**
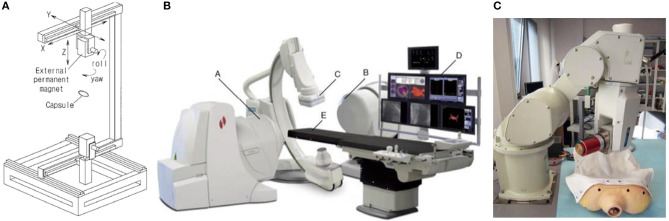
**(A)** Single permanent magnet device to perform 2-DOF rotation of the external permanent magnet through the joint unit and move the external permanent magnet in the three XYZ directions through cartesian coordinate robot (Kim et al., 2008) **(B)** Stereotaxis Niobe magnetic control system, including two independently rotating permanent magnets (Carpi and Pappone, 2009b) **(C)** External permanent magnet control system in which permanent magnet is moved by a mechanical arm with 6-DOF (Ciuti et al., 2010a).

In 2005, Kim et al. (2008) designed a capsule endoscope control system, which was controlled by a single external permanent magnet. This system has one capsule endoscope, a 2-DOF rotator, a sensor, a mechanical rod that moves at a right angle, an operating platform, and an external computer control platform. The permanent-magnet steering device is shown in Figure 5A (Kim et al., 2008). The magnetic field generated by the external permanent magnet acts on the permanent magnet in the capsule, and reflects the received magnetic force and space through the Hall sensor signal transmitted back. By moving the mechanical rod, the external permanent magnet can be moved in three directions relative to the human body. The external permanent magnet also can be rotated around the axis by a 2-DOF rotator. An external computer control platform is used to estimate the capsule direction and determine the path, thereby controlling the movement and rotation of the external permanent magnet. The external magnetic field control system can carry out unlimited movement and rotation of the capsule endoscope. At the same time, researchers pointed out that using this system for magnetic control can enable the free capsule endoscope to advance in the form of jitter.

In 2009, Stereotaxis, Inc., St. Louis, MO (Carpi and Pappone, 2009b) produced a magnetic field control system called Stereotaxis Niobe. This system has two large permanent magnets and the operating table is located between the two permanent magnets. A fluoroscope scanner is installed above the operating table, and a monitoring screen is installed on one side. Two permanent magnets are installed on the controllable robotic arm, and the control axis is on the same straight line. Stereotaxis Niobe magnetic control system can be seen in Figure 5B (Carpi and Pappone, 2009b). Using digital imaging technology, the position of micro-devices in the body can be observed on the monitoring screen in real time. The two permanent magnets can independently rotate by themselves without limitation in the rotation angles, and a relatively uniform magnetic field is generated. At present, the Stereotaxis Niobe system (Carpi and Pappone, 2009a) has been used in the diagnosis and treatment of cardiovascular diseases, especially the manipulation of intravascular magnetic catheters, and is also making great efforts to the development of magnetic capsules.

In 2009, Ciuti et al. (2010b) proposed a capsule endoscopy method, which uses an external permanent magnet to achieve steering. The laboratory uses a robotic arm system to install a single permanent magnet. Compared with the sole permanent magnet device proposed by Kim et al. (2008), this robotic arm has 6 degrees of freedom and can more effectively move the permanent magnet. Doctors need to use the human-machine interface (HMI) to accurately control the mechanical arm, displaying the actual examination on the screen to provide medical feedback. The overall control platform of the magnetic manipulation robot is shown Figure 5C (Ciuti et al., 2010a). In the next paper, Ciuti et al. (2010a) also conducted animal experiments, which further proved the superiority of this magnetic control system and improved the clinical feasibility. However, this method still has flaws that cannot be ignored. Firstly, due to the size of the camera in the capsule, the volume of the capsule will increase, affecting the swallowing and controlling work. Secondly, after the capsule entering the body, it needs a small driving force to exercise.

**Figure 6:**
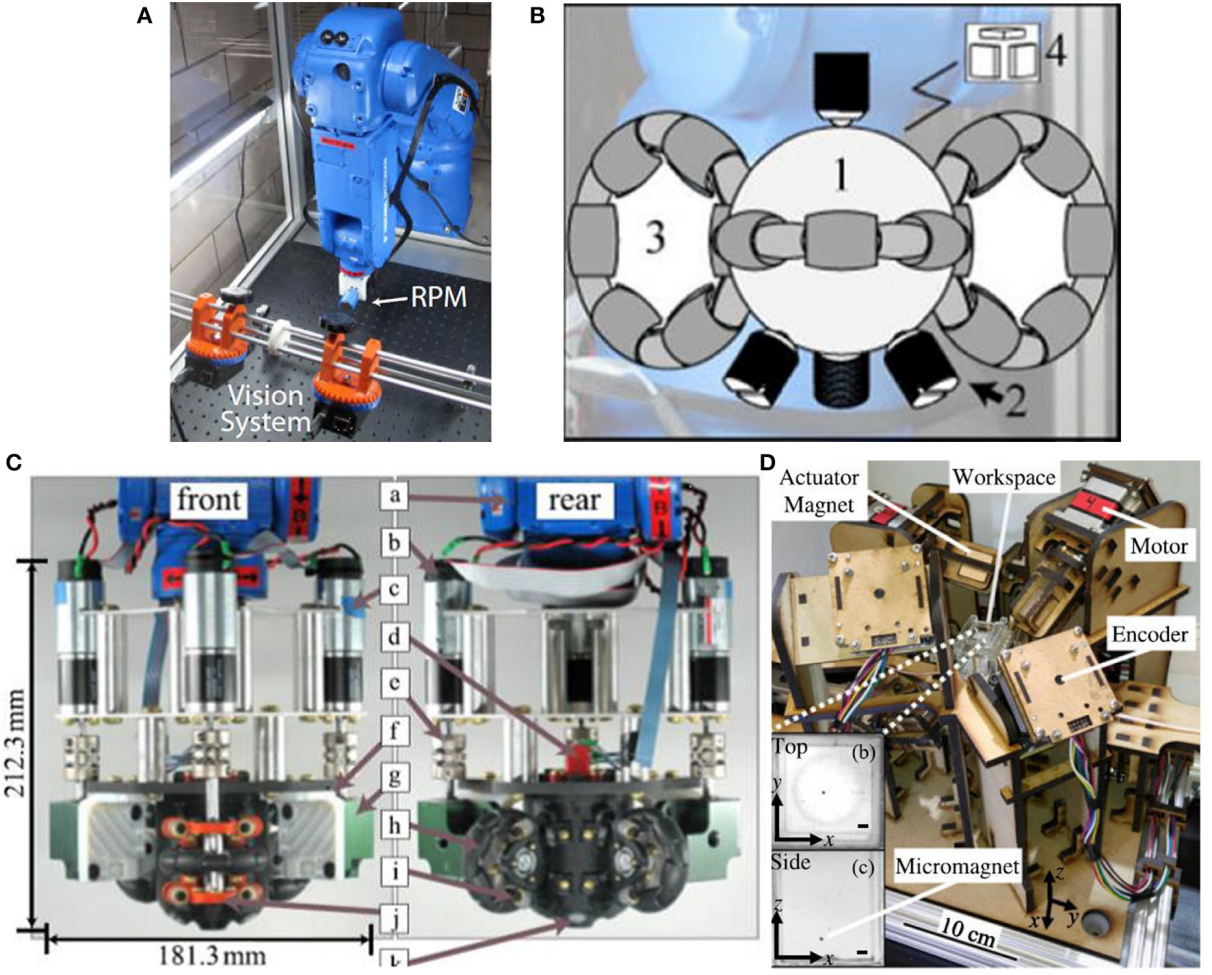
**(A)** Single rotating permanent magnet control system that rotates around a fixed rotation axis (Mahoney and Abbott, 2012) **(B)** Main configuration of magnetic ball in SAMM, 4 ball screws prevent the magnetic ball from moving and three omniwheels control the magnetic ball to rotate freely (Wright et al., 2017) **(C)** Prototype of SAMM system as the end-effector is mounted mounted to the tool frame of robotic (Wright et al., 2017) **(D)** Eight independently rotating permanent magnets control system (Ryan and Diller, 2017).

In 2012, Mahoney and Abbott (2012, 2013, 2016) and Popek et al. (2013) introduced a capsule endoscope that uses a single rotating permanent magnet for 3D manipulation. When a permanent magnet rotates around a fixed axis, each point in space has its own magnetic field vector and rotates around its own fixed axis at the same time. Built on the above, the researchers chose a single rotating permanent magnet to generate an uneven magnetic field. Most of the aforementioned single rotating permanent magnet (RPM) need to restrict magnetically actuated tools (MAT) to two fixed positions in the radial and axial directions. It will bring MAT a working dead end and limit the physical location of the PRM, which may collide with the patient, resulting in clinical harm. The non-uniform magnetic field will generate a magnetic force on the device, allowing PRM to brake at any position, and MAT can also be operated in any position. In practical applications, the position of the PRM must be adapted to the movement position of the MAT to maintain synchronization. The magnetic field generated by the PRM is modeled by a point-dipole model at the position vector of the MAT relative to the PRM, and the magnetic field matrix can be obtained. Since the matrix is reversible and the rotation axis of MAT actuation can be given, through a series of formula calculations, researchers can use this reverse solution to get the fixed rotation axis of the PRM. This closed loop method can determine the direction of the PRM at any location. This single rotating permanent magnet control system is shown in Figure 6A (Mahoney and Abbott, 2012). This makes this system break the limitation that MAT can only be rotated in a fixed direction, so that the MAT can be manipulated at any position to work.

In previous permanent magnet control systems, the problem of motion singularity is prone to occur (Kim et al., 2008; Carpi and Pappone, 2009a,b; Ciuti et al., 2010a,b; Mahoney and Abbott, 2012, 2013, 2016; Popek et al., 2013). Because the permanent magnets rely on the movement of the robot arm, there will be joint freedom and working space limitations. In 2017, Wright et al. (2017) proposed a spherical permanent magnet braking system, which can remotely control various magnetic micro-robots with five degrees of freedom. Three omniwheels with central axes orthogonal to each other can control a spherical permanent magnet to rotate in any directions. Since the permanent magnet is spherical, the generated dipole field is symmetrical about the radial axis, and there is no main inertial direction. Each position can maintain isotropy, which can perfectly address the problem of motion singularity. The prototype of the main body spherical permanent magnet is shown in Figure 6B (Wright et al., 2017). The hybrid EKF algorithm is now used to linearize this non-linear system, and then Kalman filtering is performed through a series of interference measurements to estimate the state of the dynamic system for closed loop control. The following figure shows the overall assembly prototype, with a total length of 212.3 mm and a total width of 181.3 mm, as shown in Figure 6C (Wright et al., 2017). The SAMM system can effectively demonstrate the remote control of the micro-robot in the lumen, and prove that the singularity of movement and the limitation of working space are basically eliminated.

In 2017, Ryan and Diller (2017) proposed a new magnetic control system. The system uses eight rotating permanent magnets to control the micro-robot remotely and accurately in five degrees of freedom. And it can increase the magnetic field strength and gradient strength, and generate minimum heat, as shown in Figure 6D (Ryan and Diller, 2017). The system only needs to control the external permanent magnets for rotational movement without moving parts, which can be safer and simpler. The system consists of eight permanent magnets and each permanent magnet can independently rotate from each other, so as to control the movement of the micro-robot in three-dimensional space. The motor can be placed away from the magnet, so that the heat in the working space can be reduced. Not only that, Ryan and Diller also proposed that the angular position of each magnet can be set so that the magnetic field and magnetic field gradient can be 0, which is equivalent to turning off the external magnetic field to solve the shortcomings of the previous permanent magnet systems. The system weighs the difference between the magnetic field and the force measurement unit, and uses the gradient descent method to find the corresponding local minimum value for feedback control. This driving system with 8 rotating permanent magnets can achieve a high level of control even in the case of limited working space or strictly restricted positions. However, it is inevitable that this system cannot drive high-frequency magnetic fields and cannot arbitrarily turn off the magnetic field throughout the workspace.

For permanent magnet control systems, the magnetic field can be produced without input power, thereby avoiding heat generation in the working environment. As the source of the external magnetic field, the permanent magnet can generate greater magnetic field strength and gradient strength, which can increase the efficiency of controlling the work of the robot. Moreover, it can generate enough magnetic field strength within short distance between the field source and the controlled object, and the flexibility is strong (Yang and Zhang, 2020b). However, although the mobile permanent magnet can move in a larger workspace, the strength of the magnetic field generated by the permanent magnet itself is constant. To change the magnetic field, it is necessary to accurately design the permanent magnet movement route and complex algorithms. Not only that, permanent magnet systems are generally limited by the degree of freedom of the joint of the robot arm, so the flexibility is affected. Moreover, the permanent magnet cannot turn off the field source, meaning that it is always in the “on” state, which has certain safety risks. Permanent magnets may experience magnetic field attenuation, which can cause harm to patients and medical personnel (Sendoh et al., 2002).

### Mobile Electromagnet Control Systems

**Figure 7:**
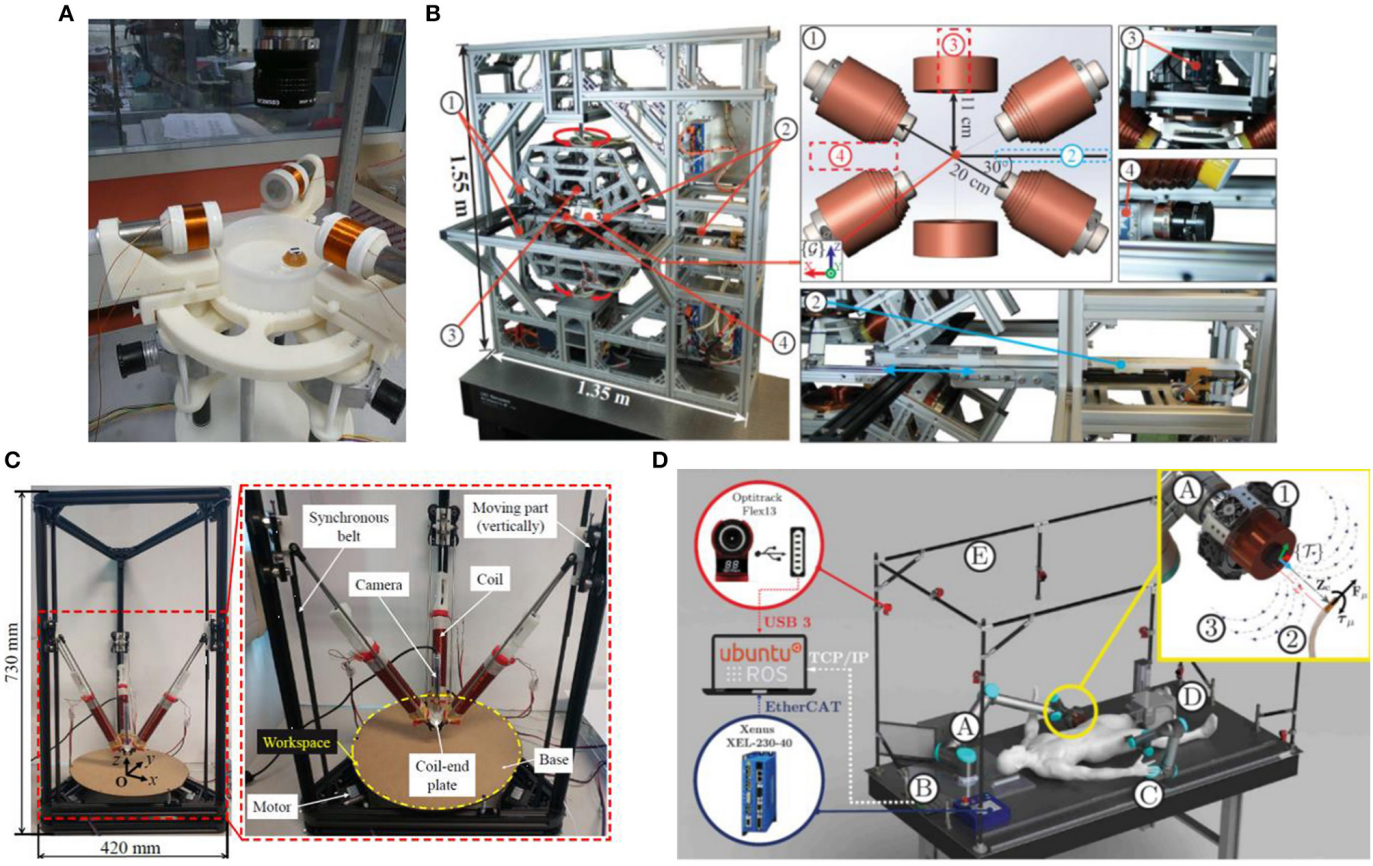
**(A)** A magnetic system consisting of 3 hollow-core coils that can rotate around their respective vertical axes (Véron et al., 2013a) **(B)** BigMag control system, including three movable and three stationary electromagnetic coils (Sikorski et al., 2017) **(C)** DeltaMag control system, including three symmetrically distributed moving coils (Yang et al., 2019); **(D)** ARMM control system in which a single cored coil is moved by a mechanical arm with 6-DOF (Sikorski et al., 2019b).

In 2013, Véron et al. (2013a) proposed a unique electromagnetic control system. Unlike the preceding stationary electromagnetic coils, movable coils combination was used in this kind of systems. The purpose is to combine some of the benefits of the above-mentioned stationary electromagnet system and moving permanent magnets, increasing the flexibility of the system to make easier control. This system consists of three movable hollow-core coils, each capable of rotating around a vertical axis. Each coil can independently control its peculiar direction and current, and measure the position of the capsule through the camera. The prototype of the system is shown in Figure 7A (Véron et al., 2013a). The micro-robot can be controlled in a plane. However, researchers have discovered that when manipulating the movable coils for magnetic field control, there will be movement singularities. In November of the same year, Véron et al. (2013b) published another paper to conduct a more in-depth study. It is known that this system is prone to meet singularity, and due to insufficient model conditions, the singularity is not easy to be detected. Researchers chose to list the electromagnetic manipulation matrix by calculation, compare and calculate the angle between the columns to simulate the conversion relationship between the current and magnetic force. These angles can be used to identify the singularity of the trajectory, so as to improve the control of the mobile electromagnet control system in order to better plan the trajectory. Nevertheless, this system can only make the micro-robot work in 2D space.

In 2017, Sikorski et al. (2017, 2019a) proposed a 3D magnetic control system, including three movable and three stationary electromagnetic coils, called BigMag. The difference between BigMag and the movable coils system proposed by Véron et al. (2013a,b) in 2013 is that it breaks the limitation of planar motion and enables micro-robots to move in three-dimensional space. Array of three mobile coils is mounted on two symmetrical moving frames, each frame containing three coils. According to the function, the coils are divided into stepping coils and ring-shaped non-stepping coils. The former has an iron core and the latter has an empty core. Each coil can rotate around its working space, and we can independently control the magnetic field generated by each coil to form an uneven magnetic field. In addition to mobile coils, BigMag device also has automatic inserters and cameras. The frame system is shown in Figure 7B (Sikorski et al., 2017), with 5-DOF control in the 3D manipulation space. It can model each coil, and design a mathematical model to grasp the magnetic field as well as coils' position changes at each location in the workspace, thereby accurately controlling the micro-robot. However, the experiment also shows that when the system inputs a higher current, some non-linear condition will appear, which is a problem that needs to be solved in the next step.

Yang et al. (2019, 2020c) designed a mobile electromagnetic control system capable of moving coils in parallel, controlling the micro-robot to brake with six degrees of freedom in three-dimensional space, which is called DeltaMag system. Three coils in DeltaMag are symmetrically distributed and can generate any magnetic field in three-dimensional space, as shown in the Figure 7C (Yang et al., 2019). DeltaMag also has a camera positioned on the plate, which can track the micro-robot in real time. Three coils are controlled in closed loop based on computer vision. It is also necessary to establish accurate mathematical modeling of each coil, and the movement of the micro-device changes with the parallel mechanism. Every time the micro-robot moves, the magnetic field needs to be updated again, so planning the movement of DeltaMag is very important. They also proposed a triple-loop visual servoing scheme, which is used to achieve both mechanism tracking and swimmer steering. The advantage of DeltaMag is that it can achieve high accurate magnetic manipulation in large workspace with small current, which can save energy. This is because it can move to the vicinity of the micro-robot and move along with it. This system shows excellent potential for gastrointestinal endoscopy and vascular catheter testing.

Sikorski et al. (2019b) proposed a magnetic field control system, which mainly conducts non-contact manipulation of *in-vivo* micro-robots, and is simply referred to as the ARMM system. The main body of the ARMM system is a single cored coil, which is fixed on a robot arm that can move with 6-DOF. The system provides a large spherical working space with a radius of 1.3 meters. At the same time, it can provide a large magnetic field and can be shut off in time to ensure safety. The ARMM magnetic control system is shown in Figure 7D (Sikorski et al., 2019b). According to the maximum load capacity of the robot arm in the ARMM system, the size of the electromagnetic coil must be designed within certain limitations. Researchers design the electromagnetic coil through the function optimization, so that the coil mass and volume are optimized within limitations, and the magnetic field can be maximized. Using Hall sensors combined with real-time updated iterative maps to solve the iron core saturation problem, and through error experiments, the system can still accurately accomplish the work in the coil saturation state. At the same time, this non-linear iterative technique can also calculate the field strength and magnetic field gradient required by the generation system to update the current input and better control the micro-robot.

In the development process, we pointed out that when a larger working space is required, if the stationary coils are redesigned, the coil size needs to be increased and the weight increased, which will increase the burden and cost of the system. Researchers found that the combination of electromagnetic coils can break the limitations of the above problems, trying to combine the stationary electromagnet control system with the permanent magnet control to form mobile electromagnet control system (Sikorski et al., 2019b). The mobile electromagnet control system tries to absorb the advantages of the above two systems to avoid shortcomings, with sufficient flexibility, and ability of turning on/off magnetic field. Not only that, compared with the stationary electromagnet system, the mobile electromagnet system can shorten the distance from the field source to the controlled micro-robot, while reducing the heat generation in the workspace. However, it is still a challenge to design the precise movement route of the mobile electromagnet system.

Contrary to the above methods, we partially change the SAMM system (Mahoney and Abbott, 2013). The bearing seat, as an example, is changed to vertical, the motor is changed to 28 stepping motors, and the structure size and volume are reduced. This change is capable to make it better used in laboratory microscope, observe the motion of the micro-robot on lens, and explore the deficiency of magnetic control system under motion, which is conducive to ameliorate it in the next step. The explicit method is described in Proposed method.

## Proposed Method

Based on the summary and analysis of the above research status, we are extremely interested in the spherical permanent magnet control system proposed by Wright et al. (2017). We believe that the SAMM system can not only generate a strong magnetic field, but also solve the problem of singularity of motion with broad prospects. In the SAMM prototype, the overall height of the system is 212.3 mm. Because SAMM is permanent magnet control system, it can generate large magnetic field strength when the object is nearby. It can also be easy to model and control without using large current without cooling problems or other maintenance issues. Therefore, these advantages make this system more suitable in the bench top experiment. In order to better analyze the motion of the micro-robot under the laboratory microscope, we have made a few design changes to the system. By shrinking the SAMM system, it is more convenient to control the motion of the micro-robot under the microscope and analyze the trajectory so that better pay attention to issues such as control accuracy and path planning. Moreover, accurate observations can also help optimize algorithms and improve control accuracy and stability. This can lay a solid foundation for the subsequent clinical trials. At this time, the spherical permanent magnet control system is in an inverted state, and the overall height must not exceed 150 mm. So as to make the permanent magnet as close as possible to the controlled micro-robot, it is necessary to ensure that the cover above the mechanism is reduced. In addition, the Hall sensors must be not too close to the permanent magnet, otherwise the reading will overflow and signals cannot be read effectively. We use SolidWorks2020 software to partially change and model the SAMM system. Since the main body of the article is a review, no specific experiment was conducted. Based on the software design, we believe that the system after design changes can be more adapted to the operation of the microscope system without affecting the control performance, and has more control stability.

### Design Details

**Figure 8:**
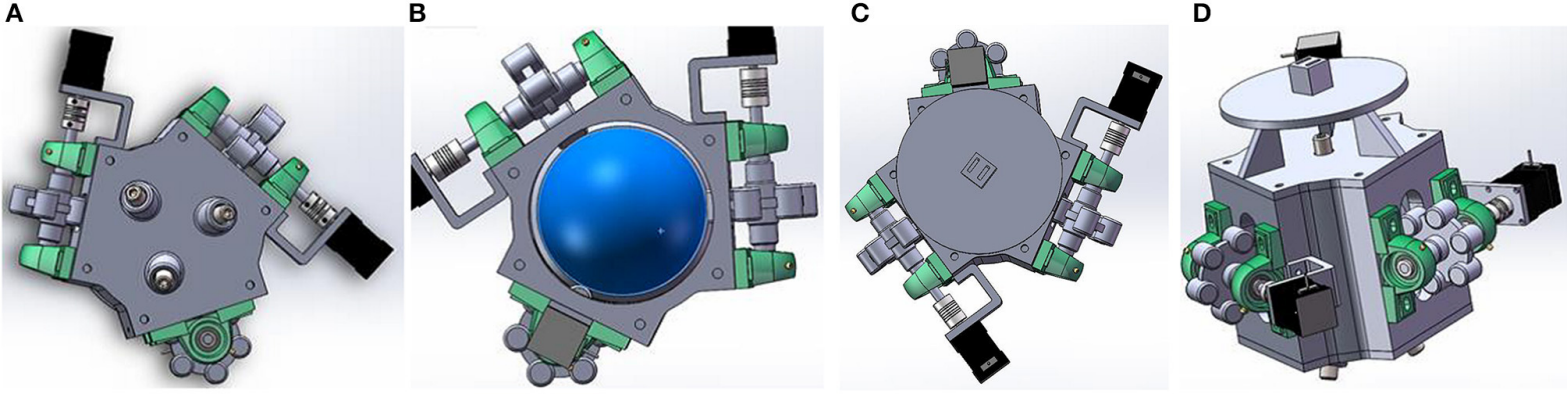
**(A)** Top view of upper cover; **(B)** Cross-section of main structure; **(C)** Bottom view of lower cover; **(D)** 3D structure of design.

In order to ensure the permanent magnet close to the robot system, we simplified the upper cover part and designed the Hall sensors under the permanent magnet. The upper cover is only composed of a detachable plate and three ball screws. The total length of the ball screws is 10 mm, which can be screwed into the main structure. The three balls are tangent to the magnetic ball on a plane. The top view of the upper cover is shown in Figure 8A.

The outside of the structure's main body has three planes with the same angle, and can provide bearing surfaces for three wheels with a diameter of 25 mm and six vertical bearing seats. Three external wheels are tangent to the magnetic ball. The tangent points of the three wheels of this design are equally divided into three sections. Since one of the wheels is still placed horizontally, it can control the spherical permanent magnet to rotate in any direction according to the speed tangential difference. The cross-section of the core structure is shown in Figure 8B. On the other side of the central axis of the wheel, a 28 stepper motor is connected by a threaded coupling. The type of the motor is 28BYG250CK-SASSML-0071, which is small in size and suitable for the size configuration of the structure.

Since the system is at an inverted state during controlling, this requires the lower cover fixed with screws. Six Hall sensors are designed under the magnetic ball, loaded through the cube slot, and fixed on the circular tray, as a whole. A ball screw with a total length of 10 mm is also embedded in the middle of the lower cover, and cooperates with the three ball screws in the upper cover to relatively fix the magnetic ball and prevent the magnetic ball from moving in space. The bottom view of the lower cover is shown in Figure 8C.

According to the 3D modeling design, the total length of the structure is 111 mm, which can control spherical permanent magnets to achieve pointing-mode control and rotating-mode control. In addition to the permanent magnet magnetic ball, all the remaining accessories are made of non-conductive non-magnetic materials as much as possible to avoid the influence of magnetic field eddy currents. The total thickness does not exceed 150 mm and the overall volume is decreased. The movement of the micro-robot can be controlled under the microscope stage. The overall 3D structure of the design is shown in Figure 8D.

## Challenges and Open Questions

In this paper, this mechanical design is only a partial change to the SAMM system. For example, the bearing seat is replaced by a vertical type, the motor is replaced by 28 stepper motors, and the structure size and volume are reduced. These changes can make it better used in laboratory microscopes to observe the movement of the micro-robot on the lens. In this way, the deficiencies of the magnetic control system can be explored under this kind of movement and improved in the next step. However, the control method and principles are still consistent with the above paper, which cannot continue to increase accuracy.

Similarly, magnetic control systems still face some problems. Owing to the complexity of disciplines, controlling and positioning algorithms are often challenging to achieve the accuracy required for clinical use. It may cause delays, resulting in inaccurate positioning and prediction. Improving work efficiency, simply changing the working space under different requirements, and solving the singularity of motion during the movement of the robot arm also need to be considered. How to design the micro-robot more safely for working in the body, and have the safety of the appropriate volume and materials are the corresponding challenges. If it is to be safely applied in the clinic, the macro experiments in the laboratory are not sufficient. Each magnetic field control system should be verified *in vivo* as much as possible. We believe that the next step requires breakthroughs in several areas: optimizing the structural design and energy supply device of the control system, seeking a higher degree of freedom control, optimizing the control algorithm of the magnetic control system and the positioning system of the micro-robot, solving the problem of movement singularity, seeking suitable materials, and completing *in-vivo* experiments as much as possible, etc. The mobile electromagnet system is a new type, which has many aspects that can be further studied, such as the modeling of magnetic fields and the design of moving paths. We also look forward to investing more work in this system.

### Conclusion

This paper elucidates magnetic control systems, which are driving systems applied to micro-robots. We classified magnetic control systems, and reviewed some research status by three categories: stationary electromagnet control system, permanent magnet control system and mobile electromagnet control system. Through the summary and analysis of these studies, we point out the advantages and shortcomings of various systems. This paper also modifies the design of the mechanical structure of the existing spherical permanent magnet control system. Utilizing SolidWorks2020 software for 3D design, the final thickness is 111 mm, which can be applied upside down under the microscope system. After the modification of this design and the discussion of the overall magnetic control system, we will not just change the mechanical structure of existing system, but try to design new systems and control algorithm optimization in the future.

## Data Availability Statement

The original contributions presented in the study are included in the article/supplementary material, further inquiries can be directed to the corresponding authors.

## Author Contributions

YS started this work and wrote the paper together with AF, CL and ML, CL adjusted and modified part of the structure on this basis. The overall process was carried out under the supervision of WZ and JS. The funding acquisition is undertaken by CL, WZ and JS. All authors contributed to the article and approved the submitted version.

## Conflict of Interest

The authors declare that the research was conducted in the absence of any commercial or financial relationships that could be construed as a potential conflict of interest.

## Publisher's Note

All claims expressed in this article are solely those of the authors and do not necessarily represent those of their affiliated organizations, or those of the publisher, the editors and the reviewers. Any product that may be evaluated in this article, or claim that may be made by its manufacturer, is not guaranteed or endorsed by the publisher.

## References

[B1] Abbott J. J. Diller E. Petruska A. J. (2020). Magnetic Methods in Robotics. Annu. Rev. Control Robot. Auton. Syst. 3, 57–90. 10.1146/annurev-control-081219-082713

[B2] Abbott J. J. Peyer K. E. Lagomarsino M. C. Kaliakatsos I. K. Nelson B. J. (2009). How should microrobots swim? Int. J. Robot. Res. 28, 1434–1447. 10.1177/0278364909341658

[B3] Boyer T. H. (1988). The force on a magnetic dipole. Am. J. Phys. 56, 688–692. 10.1119/1.15501

[B4] Carpi F. Pappone C. (2009a). Magnetic maneuvering of endoscopic capsules by means of a robotic navigation system. IEEE Trans. Biomed. Eng. 56, 1482–1490. 10.1109/TBME.2009.2013336 19174328

[B5] Carpi F. Pappone C. (2009b). Stereotaxis Niobe® magnetic navigation system for endocardial catheter ablation and gastrointestinal capsule endoscopy. Expert Rev. Med. Devices 6, 487–498. 10.1586/erd.09.32 19751121

[B6] Chen X. Z. Hoop M. Mushtaq F. Siringil E. Hu C. Nelson B. J.. (2017). Recent developments in magnetically driven micro-and nanorobots. Appl. Mater. Today 9, 37–48. 10.1016/j.apmt.2017.04.006

[B7] Ciuti G. Donlin R. Valdastri P. Arezzo A. Menciass A. Morino M.. (2010a). Robotic versus manual control in magnetic steering of an endoscopic capsule. Endoscopy 42, 148–152. 10.1055/s-0029-1243808 20017088

[B8] Ciuti G. Valdastri P. Menciassi A. Dario P. (2010b). Robotic magnetic steering and locomotion of capsule endoscope for diagnostic and surgical endoluminal procedures. Robotica 28, 199–207. 10.1017/S0263574709990361

[B9] Diller E. Floyd S. Pawshe C. Sitti M. (2012). Control of multiple heterogeneous magnetic microrobots in two dimensions on nonspecialized surfaces. IEEE Trans. Robot. 28, 172–182. 10.1109/TRO.2011.2170330

[B10] Diller E. Giltinan J. Sitti M. (2013). Independent control of multiple magnetic microrobots in three dimensions. Int. J. Robot. Res. 32, 614–631. 10.1177/0278364913483183

[B11] Gang E. S. Nguyen B. L. Shachar Y. Farkas L. Farkas L. Marx B.. (2011). Dynamically shaped magnetic fields: initial animal validation of a new remote electrophysiology catheter guidance and control system. Circ. Arrhythm. Electrophysiol. 4, 770–777. 10.1161/CIRCEP.110.959692 21690463

[B12] Jin D. Yu J. Huang T. Duan H. Zhang L. (2016). Magnetic micro-/nanoscale swimmers: Current status and potential applications. Chin. Sci. Bull. 62, 136–151. 10.1360/N972016-00854

[B13] Kim B. K. Park J. O. Hong Y. S. (2008). Capsule Type Endoscope Control System. U.S. Patent 2008/0300458 A1.

[B14] Kortschack A. Hänßler O. C. Rass C. Fatikow S. (2003). “Driving principles of mobile microrobots for micro- and nanohandling,” in Proceedings 2003 IEEE/RSJ International Conference on Intelligent Robots and Systems (IROS 2003) (Cat. No.03CH37453) (Las Vegas, NV), 1895–1900. 10.1109/IROS.2003.1248920

[B15] Kratochvil B. E. Kummer M. P. Erni S. Borer R. Frutiger D. R. Schürle S.. (2014). “MiniMag: a hemispherical electromagnetic system for 5-DOF wireless micromanipulation,” in Experimental Robotics, eds Khatib O. Kumar V. Sukhatme G. (Berlin: Springer), 317–329. 10.1007/978-3-642-28572-1_22

[B16] Kummer M. P. Abbott J. J. Kratochvil B. E. Borer R. Sengul A. Nelson B. J. (2010). OctoMag: an electromagnetic system for 5-DOF wireless micromanipulation. IEEE Trans. Robot. 26, 1006–1017. 10.1109/TRO.2010.2073030

[B17] Lee C. S. Lee H. Westervelt R. E. (2001). Microelectromagnets for the control of magnetic nanoparticles. Appl. Phys. Lett. 79, 3308–3310. 10.1063/1.1419049

[B18] Lee H. Purdon A. M. Westervelt R. M. (2004). Micromanipulation of biological systems with microelectromagnets. IEEE Trans. Magn. 40, 2991–2993. 10.1109/TMAG.2004.829179

[B19] Li J. Li X. Luo T. Wang R. Liu C. Chen S.. (2018). Development of a magnetic microrobot for carrying and delivering targeted cells. Sci. Robot. 3:eaat8829. 10.1126/scirobotics.aat8829 33141689

[B20] Li J. Wang H. Shi Q. Zheng Z. Cui J. Sun T.. (2020). Biped walking of magnetic in oscillating field for indirect manipulation of non-magnetic objects. IEEE Trans. Nanotechnol. 19, 21–24. 10.1109/TNANO.2019.2954312

[B21] Mahoney A. W. Abbott J. J. (2012). Control of untethered magnetically actuated tools with localization uncertainty using a rotating permanent magnet, in 2012 4th IEEE RAS & EMBS International Conference on Biomedical Robotics and Biomechatronics (BioRob) (Rome), 1632–1637. 10.1109/BioRob.2012.6290306

[B22] Mahoney A. W. Abbott J. J. (2013). “5-DOF manipulation of a magnetic capsule in fluid using a single permanent magnet: proof-of-concept for stomach endoscopy,” in Hamlyn Symposium on Medical Robotics, 114–115. 10.15607/RSS.2014.X.037

[B23] Mahoney A. W. Abbott J. J. (2016). Five-degree-of-freedom manipulation of an untethered magnetic device in fluid using a single permanent magnet with application in stomach capsule endoscopy. Int. J. Robot. Res. 35, 129–147. 10.1177/0278364914558006

[B24] Monk P. (1992). Analysis of a finite element method for Maxwell's equations. SIAM J. Numer. Anal. 29, 714–729. 10.1137/0729045

[B25] Nelson B. J. Kaliakatsos I. K. Abbott J. J. (2010). Microrobots for minimally invasive medicine. Annu. Rev. Biomed. Eng. 12, 55–85. 10.1146/annurev-bioeng-010510-103409 20415589

[B26] Ng W. M. Teng X. J. Guo C. Liu C. Low S. C. Chan D. J. C.. (2019). Motion control of biohybrid microbots under low reynolds number environment: magnetotaxis. Chem. Eng. Process. Process Intensification 141:107530. 10.1016/j.cep.2019.107530

[B27] Nguyen V. D. Leon-Rodriguez H. Lee C. Zhen J. Choi H. C. Ko G.. (2016). Novel active locomotive capsule endoscope with micro-hydraulic pump for drug delivery function, in 2016 6th IEEE International Conference on Biomedical Robotics and Biomechatronics (BioRob) (University Town), 311–316. 10.1109/BIOROB.2016.7523644

[B28] Ongaro F. Pane S. Scheggi S. Misra S. (2019). Design of an electromagnetic setup for independent three-dimensional control of pairs of identical and nonidentical microrobots. IEEE Trans. Robot. 35, 174–183. 10.1109/TRO.2018.2875393

[B29] Petruska A. J. Abbott J. J. (2014). Omnimagnet: an omnidirectional electromagnet for controlled dipole-field generation. IEEE Trans. Magn. 50, 1–10. 10.1109/TMAG.2014.2303784

[B30] Popek K. M. Mahoney A. W. Abbott J. J. (2013). Localization method for a magnetic capsule endoscope propelled by a rotating magnetic dipole field, in 2013 IEEE International Conference on Robotics and Automation (Karlsruhe), 5348–5353. 10.1109/ICRA.2013.6631343

[B31] Pourkand A. Abbott J. J. (2018). A critical analysis of eight-electromagnet manipulation systems: the role of electromagnet configuration on strength, isotropy, and access. IEEE Robot. Autom. Lett. 3, 2957–2962. 10.1109/LRA.2018.2846800

[B32] Ryan P. Diller E. (2017). Magnetic actuation for full dexterity microrobotic control using rotating permanent magnets. IEEE Trans. Robot. 33, 1398–1409. 10.1109/TRO.2017.2719687

[B33] Salmanipour S. Diller E. (2018). Eight-degrees-of-freedom remote actuation of small magnetic mechanisms, in IEEE International Conference on Robotics and Automation (ICRA) (Brisbane, QLD: Brisbane Convention & Exhibition Centre), 3608–3613. 10.1109/ICRA.2018.8461026

[B34] Sarton G. Mayer J. R. Joule J. P. Carnot S. (1929). The discovery of the law of conservation of energy. Isis 13, 18–44. 10.1086/34643011611872

[B35] Schmidt C. K. Medina-Sánchez M. Edmondson R. J. Schmidt O. G. (2020). Engineering microrobots for targeted cancer therapies from a medical perspective. Nat. Commun. 11, 1–18. 10.1038/s41467-020-19322-7 33154372 PMC7645678

[B36] Sendoh M. Ishiyama K. Arai K. I. (2002). Direction and individual control of magnetic micromachine. IEEE Trans. Magn. 38, 3356–3358. 10.1109/TMAG.2002.802306

[B37] Sikorski J. Dawson I. Denasi A. Hekman E. E. G. Misra S. (2017). Introducing BigMag — A novel system for 3D magnetic actuation of flexible surgical manipulators, in 2017 IEEE International Conference on Robotics and Automation (ICRA) (Marina Bay Sands), 3594–3599. 10.1109/ICRA.2017.7989413

[B38] Sikorski J. Denasi A. Bucchi G. Scheggi S. Misra S. (2019a). Vision-based 3-D control of magnetically actuated catheter using BigMag—an array of mobile electromagnetic coils. IEEE/ASME Trans. Mechatr. 24, 505–516. 10.1109/TMECH.2019.2893166

[B39] Sikorski J. Heunis C. M. Franco F. Misra S. (2019b). The ARMM system: an optimized mobile electromagnetic coil for non-linear actuation of flexible surgical instruments. IEEE Trans. Magn. 55, 1–9. 10.1109/TMAG.2019.2917370

[B40] Sitti M. Ceylan H. Hu W. Giltinan J. Turan M. Yim S.. (2015). Biomedical applications of untethered mobile milli/microrobots. Proc. IEEE 103, 205–224. 10.1109/JPROC.2014.2385105 27746484 PMC5063027

[B41] Song S. Song S. Meng M. Q. (2017). “Electromagnetic actuation system using stationary six-pair coils for three-dimensional wireless locomotive microrobot,” in 2017 IEEE International Conference on Information and Automation (ICIA), 305–310. 10.1109/ICInfA.2017.8078924

[B42] Su M. Xu T. Lai Z. Huang C. Liu J. Wu X. (2020). Double-modal locomotion and application of soft cruciform thin-film microrobot. IEEE Robot. Autom. Lett. 5, 806–812. 10.1109/LRA.2020.2965912

[B43] Sun T. Wang H. Shi Q. Takeuchi M. Nakajima M. Huang Q.. (2016). Micromanipulation for coiling microfluidic spun alginate microfibers by magnetically guided system. IEEE Robot. Autom. Lett. 1, 808–813. 10.1109/LRA.2016.2524991

[B44] Vaidman L. (1990). Torque and force on a magnetic dipole. Am. J. Phys. 58, 978–983. 10.1119/1.16260

[B45] Véron B. Abadie J. Hubert A. Andreff N. (2013a). Magnetic manipulation with several mobile coils towards gastrointestinal capsular endoscopy. New Trends Mech. Mach. Sci. 7, 681–689. 10.1007/978-94-007-4902-3_71

[B46] Véron B. Hubert A. Abadie J. Andreff N. (2013b). Geometric analysis of the singularities of a magnetic manipulation system with several mobile coils, in 2013 IEEE/RSJ International Conference on Intelligent Robots and Systems (Tokyo), 4996–5001. 10.1109/IROS.2013.6697078

[B47] Wang Q. Yang L. Wang B. Yu E. Yu J. Zhang L. (2018). Collective behavior of reconfigurable magnetic droplets via dynamic self-assembly. ACS Appl. Mater. Interfaces 11, 1630–1637. 10.1021/acsami.8b17402 30560650

[B48] Wright S. E. Mahoney A. W. Popek K. M. Abbott J. J. (2017). The spherical-actuator-magnet manipulator: a permanent-magnet robotic end-effector. IEEE Trans. Robot. 33, 1013–1024. 10.1109/TRO.2017.2694841

[B49] Xu T. Yu J. Vong C. Wang B. Wu X. Zhang L. (2019). Dynamic morphology and swimming properties of rotating miniature swimmers with soft tails. IEEE/ASME Trans. Mechatronics 24, 924–934. 10.1109/TMECH.2019.2912404

[B50] Xu T. Yu J. Yan X. Choi H. Zhang L. (2015). Magnetic actuation based motion control for microrobots: an overview. Micromachines 6, 1346–1364. 10.3390/mi6091346

[B51] Yan X. Zhou Q. Vincent M. Deng Y. Yu J. Xu J.. (2017). Multifunctional biohybrid magnetite microrobots for imaging-guided therapy. Sci. Robot. 2:eaaq1155. 10.1126/scirobotics.aaq1155 33157904

[B52] Yan X. Zhou Q. Yu J. Xu T. Deng Y. Tang T.. (2015). Magnetite nanostructured porous hollow helical microswimmers for targeted delivery. Adv. Funct. Mater. 25, 5333–5342. 10.1002/adfm.201502248

[B53] Yang L. Du X. Yu E. Jin D. Zhang L. (2019). DeltaMag: An Electromagnetic Manipulation System with Parallel Mobile Coils, in 2019 International Conference on Robotics and Automation (ICRA) (Montreal, QC), 9814–9820. 10.1109/ICRA.2019.8793543

[B54] Yang L. Wang Q. Vong C. Zhang L. (2017). A miniature flexible-link magnetic swimming robot with two vibration modes: design, modeling and characterization. IEEE Robot. Autom. Lett. 2, 2024–2031. 10.1109/LRA.2017.2718104

[B55] Yang L. Yu J. Zhang L. (2020a). Statistics-based automated control for a swarm of paramagnetic nanoparticles in 2-D space. IEEE Trans. Robot. 36, 254–270. 10.1109/TRO.2019.2946724

[B56] Yang L. Zhang L. (2020a). Motion control in magnetic microrobotics: from individual and multiple robots to swarms. Annu. Rev. Control Robot. Auton. Syst. 4, 509–534. 10.1146/annurev-control-032720-104318

[B57] Yang L. Zhang Y. Wang Q. Chan K. Zhang L. (2020b). Automated control of magnetic spore-based microrobot using fluorescence imaging for targeted delivery with cellular resolution. IEEE Trans. Autom. Sci. Eng. 17, 490–501. 10.1109/TASE.2019.2937232

[B58] Yang Z. Yang L. Zhang L. (2020c). 3-D visual servoing of magnetic miniature swimmers using parallel mobile coils. IEEE Trans. Med. Robot. Bionics 2, 608–618. 10.1109/TMRB.2020.3033020

[B59] Yang Z. Zhang L. (2020b). Magnetic actuation systems for miniature robots: a review. Adv. Intell. Syst. 2:2000082. 10.1002/aisy.202000082

[B60] Yu J. Jin D. Chan K. Wang Q. Yuan K. Zhang L. (2019). Active generation and magnetic actuation of microrobotic swarms in bio-fluids. Nat. Commun. 10, 1–12. 10.1038/s41467-019-13576-6 31822669 PMC6904566

[B61] Yu J. Xu T. Lu Z. Vong C. I. Zhang L. (2017). On-demand disassembly of paramagnetic nanoparticle chains for microrobotic cargo delivery. IEEE Trans. Robot. 33, 1213–1225. 10.1109/TRO.2017.2693999

[B62] Yu J. Yang L. Zhang L. (2018). Pattern generation and motion control of a vortex-like paramagnetic nanoparticle swarm. Int. J. Robot. Res. 37, 912–930. 10.1177/0278364918784366

[B63] Zhang L. Abbott J. J. Dong L. Kratochvil B. E. Bell D. Nelson B. J. (2009). Artificial bacterial flagella: Fabrication and magnetic control. Appl. Phys. Lett. 94:064107. 10.1063/1.3079655

[B64] Zhang Q. Song S. Song S. (2017). Study on magnetic field model of independent circular coils for wireless manipulation of microrobots, in 2017 IEEE International Conference on Information and Automation (ICIA) (Macau), 1137–1142. 10.1109/ICInfA.2017.8079073

[B65] Zheng Z. Wang H. Dong L. Shi Q. Li J. Sun T.. (2021). Ionic shape-morphing microrobotic end-effectors for environmentally adaptive targeting, releasing, and sampling. Nat. Commun. 12, 1–12. 10.1038/s41467-020-20697-w 33462214 PMC7814140

